# Systemic TNFα correlates with residual β-cell function in children and adolescents newly diagnosed with type 1 diabetes

**DOI:** 10.1186/s12887-020-02339-8

**Published:** 2020-09-23

**Authors:** Anne Julie Overgaard, Jens Otto Broby Madsen, Flemming Pociot, Jesper Johannesen, Joachim Størling

**Affiliations:** 1 Steno Diabetes Center Copenhagen, Gentofte, Denmark; 2 Pediatrics Department E, Herlev Hospital, Herlev, Denmark; 3 Faculty of Health and Medical Sciences, University of Copenhagen, Copenhagen, Denmark; 4 Department of Biomedical Sciences, University of Copenhagen, Copenhagen, Denmark

**Keywords:** β-cell function, Cytokines, TNF, Inflammation, Type 1 diabetes, Remission

## Abstract

**Background:**

Type 1 diabetes (T1D) is caused by immune-mediated destruction of the β-cells. After initiation of insulin therapy many patients experience a period of improved residual β-cell function leading to partial disease remission. Cytokines are important immune-modulatory molecules and contribute to β-cell damage in T1D. The patterns of systemic circulating cytokines during T1D remission are not clear but may constitute biomarkers of disease status and progression. In this study, we investigated if the plasma levels of various pro- and anti-inflammatory cytokines around time of diagnosis were predictors of remission and residual β-cell function in children with T1D followed for one year after disease onset.

**Methods:**

In a cohort of 63 newly diagnosed children (33% females) with T1D with a mean age of 11.3 years (3.3–17.7), ten cytokines were measured of which eight were detectable in plasma samples by Mesoscale Discovery multiplex technology at study start and after 6 and 12 months. Linear regression models were used to evaluate association of cytokines with stimulated C-peptide.

**Results:**

Systemic levels of tumor necrosis factor (TNF)-α, interleukin (IL)-2 and IL-6 inversely correlated with stimulated C-peptide levels over the entire study (*P* < 0.05). The concentrations of TNFα and IL-10 at study start predicted stimulated C-peptide level at 6 months (*P* = 0.011 and *P* = 0.043, respectively, adjusted for sex, age, HbA1c and stage of puberty).

**Conclusions:**

In recent-onset T1D, systemic cytokine levels, and in particular that of TNFα, correlate with residual β-cell function and may serve as prognostic biomarkers of disease remission and progression to optimize treatment strategies.

**Trial Registration:**

The study was performed according to the criteria of the Helsinki II Declaration and was approved by the Danish Capital Region Ethics Committee on Biomedical Research Ethics (journal number H-3-2014-052). The parents of all participants gave written consent.

## Background

Type 1 diabetes (T1D) is the consequence of immune-mediated destruction of the insulin-producing pancreatic β-cells. A transient period of partial disease remission is seen in many T1D patients shortly after initiation of insulin therapy. This ‘honeymoon’ period is characterized by transitory restoration of endogenous insulin production, lower insulin requirement and better glycemic control [1, 2]. Prediction of partial remitters at time of diagnosis may facilitate optimized and individualized patient care.

The pathogenesis of T1D is multifactorial and not fully understood. Cytokines are important regulators of immune and inflammatory processes and specific cytokines such as interleukin (IL)-1β and tumor necrosis factor (TNF)-α released by intra-pancreatic immune cells contribute to T1D by directly causing β-cell impairment [3, 4]. Systemic plasma levels of cytokines in new-onset T1D may reflect the detrimental events occuring in the pancreas and might predict disease remission and/or be biomarkers of disease status. Accordingly, a recent study suggested an association between immune cell subtypes and serum cytokine patterns with metabolic control in children newly diagnosed with T1D [5].

In this study, we investigated the correlation between ten systemic cytokines and metabolic deterioration in children with recent-onset T1D to predict disease course during first year after diagnosis.

## Methods

### Study subjects

Blood samples obtained from a total of 63 children and adolescents with newly diagnosed type 1 diabetes (T1D) based on the ISPAD (International Society for Pediatric and Adolescent Diabetes) criteria for diagnosis defined as the first visit with high blood glucose levels and initiation of insulin treatment as described by Madsen et al. [6]. The mean age at study start was 11.3 (3.3–17.7). Mean time from diagnosis to first visit and, hence, baseline sample was 2.45 ± 0.65 months. At each visit a physician examined wether the participants had entered puberty according to the Tanner criteria [7, 8]. Pubertal stages were then categorized as prepubertal (Tanner 1) or pubertal (Tanner 2-5). Parents or guardians all gave written informed consent. The study was performed according to the criteria of the Helsinki II Declaration and was approved by the Danish Capital Region Ethics Committee on Biomedical Research Ethics (journal number H-3-2014-05). Plasma samples from first visit after being enrolled in study (baseline), and repeated 6 and 12 months later were used in this study.

### Residual β-cell function and HbA1c

The endogenous insulin production in the participants was assesed by measuring stimulated serum C-peptide levels at study start (defined as baseline) and 6 and 12 months after inclusion. Blood was drawn at fasting and 90 minutes after ingestion of a standardized liquid mixed-meal (Boost High Protein, Nestlé Health Science, or Ensure Plus, Abbott Nutrition). Plasma and serum were isolated accordingly and samples were stored at − 80˚C until analysed. C-peptide and HbA1c were measured using an Immulite® 2000 chemiluminescent immunometric assay and high-pressure liquid chromatography (Tosoh Bioscience, South San Francisco, CA, USA), respectively.

### Cytokine quantifications

Cytokine concentrations in fasting plasma samples were measured using the V-PLEX Proinflammatory Panel 1 Human Kit (Meso Scale Diagnostics® [MSD], Gaithersburg, MD, USA) quantifying interferon (IFN)-γ, interleukin (IL)-1β, interleukin-2 (IL-2), interleukin-4 (IL-4), interleukin-6 (IL-6), interleukin (IL-8), interleukin (IL-10), interleukin-12p70 (IL-12p70), interleukin-13 (IL-13) and tumor necrosis factor (TNF)-α according to the manufacturers instructions using the MESO QuickPlex SQ120 instrument. Analytes below the detection limit of the assay and analytes with a coefficient of variation (CV) > 30% were discarded for further analysis and included IL-1β and IL-13. Plate-to-plate variation was assessed by including identical pooled plasma samples and the CV between plates for each analyte was 8-25.8%.

### Statistical analysis

Variance in clinical and anthropometrics data between time points were assessed using ANOVA with a Tukey post hoc test. Associations between cytokine levels and stimulated C-peptide levels were evalutaed using linear regression. Linear regression models adjusted for sex, age, HbA1c and puberty status according to the Tanner criteria were constructed using cytokine levels at baseline as predictors of stimulated C-peptide levels at 6 and 12 months as outcome. We did not include BMI as a covariate because of the strong correlation between age and BMI (Pearson correlation: r = 0.6093, *P* < 0.0001). C-peptide levels were log transformed before statistical analysis. All statistical tests were performed using GraphPad Prism8 or the free statitistical environment R (https://www.r-project.org/).

## Results

### Correlation of cytokine levels with stimulated C-peptide

**Fig. 1:**
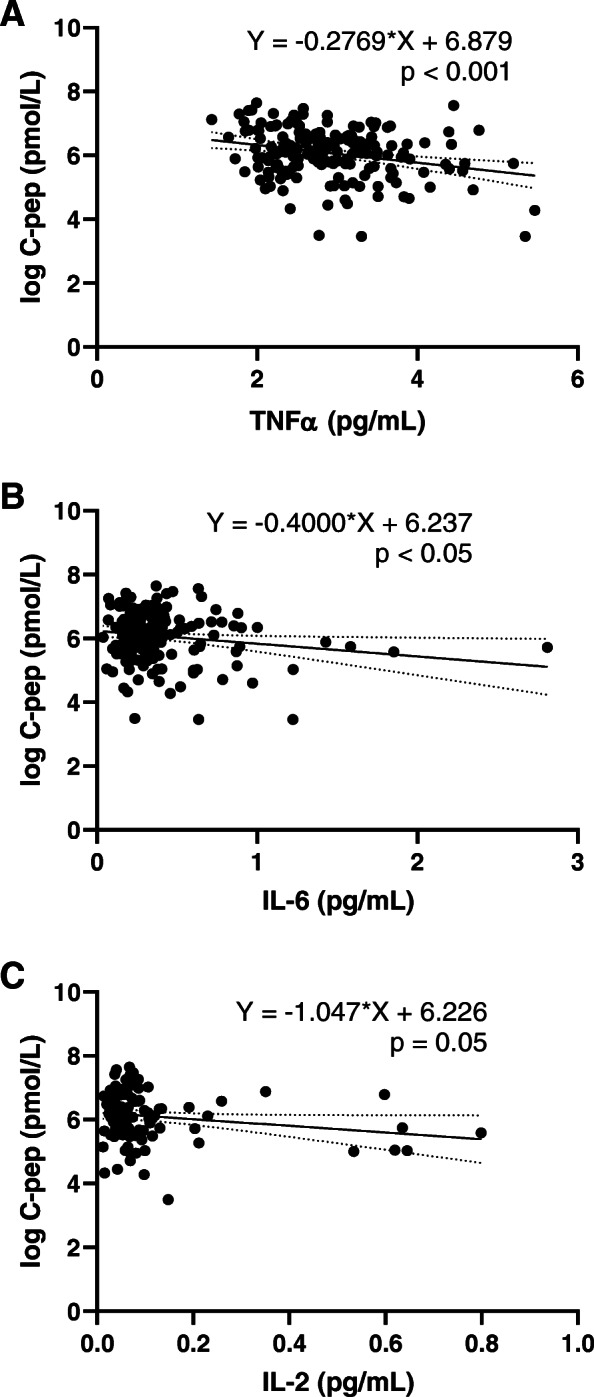
Correlation of cytokines and stimulated C-peptide levels in newly diagnosed children and adolescents with type 1 diabetes. Linear regression models between cytokine levels and stimulated C‐peptide levels the first year after T1D onset. C-peptide levels were log transformed before analysis

The clinical characteristics of the study participants and the cytokine concentrations measured are shown in Table 1. We first determined the fluctuation of the eight measurable cytokines over time. No significant differences in the concentrations of any of the cytokines between baseline and 6 and 12 months were observed (Table 1). However, the levels of TNFα, IL-2 and IL-6 correlated negatively with stimulated C-peptide levels (Fig. 1).

**Table 1:** Clinical characteristics and cytokine concentrations of study population at the time of blood sampling

	Baseline visit	Visit 6 months	Visit 12 months
	Mean (SD)	Mean (SD)	Mean (SD)
N (% females)	63 (33)	63 (33)	63 (33)
Age (years)	11.3 (3.3–17.7)	11.8 (3.8–18.2)	12.4 (4.4–18.7)
HbA1c (mmol/mol)	51.73 (11.1)	49 (8)	53.05(13.4)
C-peptide, fasting (pmol/L)	194.89 (118.5)	172.46 (114.7)	148.95 (121.4)
C-peptide, stimulated* (pmol/L)	685.54 (377.5)^@#^	518.3 (364.3)	446.59 (377.5)
BMI (kg/m^2^)	17.96 (2.8)	18.43 (3)	18.75 (3.1)
BMI-SDS	-0.03 (1)	0.07 (1)	0.07 (1)
IFN-γ (pg/mL)	1.87 (2.05)	2.15 (5.08)	2.36 (5.2)
IL-10 (pg/mL)	0.63 (0.3)	0.56 (0.2)	0.57 (0.3)
IL-12p70 (pg/mL)	0.15 (0.1)	0.13 (0.1)	0.14 (0.1)
IL-2 (pg/mL)	0.12 (0.2)	0.1 (0.1)	0.09 (0.1)
IL-4 (pg/mL)	0.05 (0.0)	0.04 (0.0)	0.04 (0.0)
IL-6 (pg/mL)	0.42 (0.4)	0.4 (0.3)	0.36 (0.3)
IL-8 (pg/mL)	4.37 (1.7)	4.03 (0.9)	3.91 (1.1)
TNFα (pg/mL)	3.03 (0.7)	2.92 (0.7)	2.8 (0.8)

Characteristics at time of blood sampling. Data is presented as mean (SD) or mean (range) where indicated. *BMI *Body mass index, *BMI-SDS *BMI standard deviation score, *HbA1c *Haemoglobin A1c. *P < 0.05 in comparison between groups evaluated using ANOVA. @*P* < 0.05 between baseline and 6 months #*P* < 0.05 between baseline and 12 months ¤*P* < 0.05 between 6 months and 12 months

### Cytokine levels at baseline as predictors of stimulated C-peptide

Throughout the study period, most study participants were in partial remission as defined by a stimulated C-peptide level > 300 pmol/L. The highest mean stimulated C-peptide level was at baseline and declinded gradually at 6 and 12 months (Table 1). Baseline levels of IL-10 (intercept − 0.57 (confidence interval (CI): -1; -0.1)) and TNFα (intercept − 0.38 (CI: -0.6; -0.1)) individually predicted lower stimulated C-peptide at 6 months (*P* = 0.043 and *P* = 0.011, respectively, adjusted for sex, age, HbA1c and Tanner criteria) (Table 2).

**Table 2:** Linear regression modelling of cytokine levels at study start (baseline) and stimulated C-peptide (log transformed) at 6 and 12 months

Baseline cytokines association to:	C-peptide at 6 months, adjusted for sex, age, HbA1c and Tanner		C-peptide at 12 months adjusted for sex, age, HbA1c and Tanner	
Predictor	Outcome Intercept CI (5–95%)	*P*-value	Outcome Intercept CI (5–95%)	*P*-value
IFN-γ	-0.03 (-0.1; 0)	0.507	0.03 (0; 0.1)	0.451
IL-10	-0.57 (-1; -0.1)	**0.043**	0.23 (-0.2; 0.7)	0.384
IL-12p70	-1.85 (-3.8; 0.1)	0.122	-1.23 (-2.7; 0.2)	0.165
IL-2	-0.66 (-1.8; 0.5)	0.333	0.3 (-0.8; 1.4)	0.640
IL-4	-10.3 (-19.5; -1.1)	0.069	6.64 (-4.8; 18.1)	0.328
IL-6	-0.38 (-0.77; 0)	0.117	0.14 (-0.2; 0.5)	0.514
IL-8	0.03 (-0.1; 0.2)	0.620	-0.05 (-0.2; 0.1)	0.535
TNFα	-0.38 (-0.6; -0.1)	**0.011**	0 (-0.2; 0.2)	0.976

## Discussion

The partial disease remission seen in many T1D patients within a few months after diagnosis may be a possible target checkpoint for future therapeutic interventions aiming at preserving residual β-cell mass. It may also be used to identify individuals with a lower or higher risk of developing long-term complications. Identification of specific biomarkers of disease remission are therefore important to facilitate optimized treatment strategies.

We measured the systemic levels of ten cytokines in a cohort of children newly diagnosed with T1D. There were no differences in the mean concentrations of any of the eight measurable cytokines over the entire study period indicating intra-individual stability of cytokines over time. The concentrations of TNFα, IL-2 and IL-6, however, associated inversely with stimulated C-peptide. Further, the levels of IL-10 and TNFα at baseline predicted lower stimulated C-peptide at 6 months. That TNFα turned out significant in both analyses suggest that TNFα may be a particularly important determinant of residual β-cell function in newly diagnosed T1D patients.

TNFα is well-known to exert detrimental effects on β-cells *in vitro* especially when combined with other cytokines such as IL-1β and/or IFN-γ, and are believed to contribute to β-cell destruction in T1D [3]. A small placebo-controlled clinical trial investigating the effect of a 24-week therapy with etanercept, an anti-TNF agent used to treat rheumatoid arthritis, in children newly diagnosed with T1D reported an increase in C-peptide and a decrease in HbA1c in the etanercept group [9]. These results suggest that targeting TNFα early in T1D can boost and possibly prolong the honeymoon phase in T1D via improved β-cell function. The observed inverse correlation between TNFα plasma levels and stimulated C-peptide during the remission period may help stratify patients for anti-TNF therapy in future studies.

In line with our findings, a previous study of T1D remission in adults found that the levels of circulating cytokines including IL-10 at time of diagnosis are predictive of clinical remission [10]. Another study of T1D remission in children also found an inverse correlation between serum IL-10 and C-peptide [11]. Association between IL-10 and C-peptide in recent-onset T1D therefore seems strong and IL-10 may therefore be a robust inverse biomarker of residual β-cell function.

The pathogenic role of IL-10 in T1D is unclear. IL-10 is an anti-inflammatory cytokine that dampens the activity of antigen-presenting cells and T helper and cytotoxic T cells [12]. Our finding that IL-10 is inversely associated with stimulated C-peptide therefore seems counterintuitive. However, as IL-10 may also possess pro-inflammatory effects depending on the exact biological setting [13], it cannot be excluded that IL-10 has a dual role in T1D. No human trials administering or targeting IL-10 have so far been reported, but studies using the non-obese diabetic (NOD) mouse model of T1D found that IL-10 has protective effects on diabetes development [14, 15]. Irrespective of the precise pathophysiological role of IL-10 in T1D, our results suggest that measurement of plasma IL-10 around time of diagnosis may be used to predict remitters and the speed of decline in residual β-cell function.

A major limitation of our study is the limited number of study participants which hamper the statistical power. Hence, larger studies with more power are warranted and will likely provide more information about the relationship between systemic inflammatory factors and partial remission. Another drawback of our study is that the baseline plasma samples were taken on average 2.45 months after T1D diagnosis. The reason for this is that the samples originally were obtained with the purpose of studying bone markers at the peak of remission. Therefore, at this time point, most of the patients had the highest level of residual β-cell function reflecting maximal partial remission. It would have been preferable to have the baseline samples taken closer to the time of diagnosis to see if the levels of cytokines at that time could predict the peak amplitude of remission.

## Conclusions

In summary, our results show an inverse correlation between certain cytokines and residual β-cell function in children and adolescents with newly diagnosed T1D. The level of two of the cytokines, i.e. TNFα and IL-10, at time around diagnosis are predictors of β-cell function after 6 months and may thus be exploited as biomarkers to better monitor and predict clinical remission. As TNFα was both a prognostic marker of residual β-cell function and was negatively associated with C-peptide levels throughout the study period highlight TNFα as the main finding of this study. Whether TNFα (and IL-10) plays a causative role in disease remission or merely associate with remission remains unclear.

## Data Availability

The datasets used and/or analysed during the current study are available from the corresponding author on reasonable request.

## Abbreviations

BMI: Body mass index
HbA1c: Hemoglobin A1c
IFN: Interferon
IL: Interleukin
MSD: Meso Scale Discovery
T1D: Type 1 diabetes
TNF: Tumor necrosis factor
